# Gastrodin improves learning behavior in a rat model of Alzheimer's disease induced by intra-hippocampal Aβ 1-40 injection

**DOI:** 10.1186/1750-1326-7-S1-S15

**Published:** 2012-02-07

**Authors:** Xing Liu, Mengya Wang

**Affiliations:** 1 Cell Electrophysiology Laboratory, Wannan Medical College, Wuhu, Anhui 241002, China

## Background

Gastrodin extracted from the rhizome of *Gastrodia elata* Blume, a Chinese herbal medicine, has long been used for treating vertigo, general paralysis, epilepsy, tetanus, stroke and dementia. Although several reports have shown that gastrodin has neuroprotective effects to rescue lead-induced synaptic plasticity deficits in rat hippocampus, and hippocampal cell damage in cellular model of Alzheimer’s disease induced by Aβ25*-*35, its effect on behaviors of rat model of Alzheimer's disease induced by intra-hippocampal Aβ injection is not studied yet.

## Methods

Forty-one male adult Sprague–Dawley rats were randomly divided into groups: normal (*n*=10), sham operated (*n*=7, intra-hippocampal saline injection), saline (*n*=8, intra-hippocampal Aβ injection and then ig saline), gastrodin (*n*=8, intra-hippocampal Aβ injection and then ig gastrodin), huperzine A (*n*=8, intra-hippocampal Aβ injection and then ig huperzine A). One week after intra-hippocampal Aβ1-40 injection (5 μg in 1 μl PBS, bilaterally), gastrodin (200 mg/kg), huperzine A (300 μg/kg) or saline were administrated by ig for 27 days (*q.d.*). At end of gastrodin or huperzine A treatment, the 5-day Morris water maze test was performed to observe the learning and memory function of 5 groups of animals.

## Results

Two-way ANOVA (repeated measures) was used to compare the difference of escape latency in place navigation trials among testing days or groups, and showed both differences among 5 test days and 5 groups were very significant (*P*<0.001). Bonferroni posttests indicated that the difference between normal and sham operated was not significant (*P*>0.05), but the latency in saline group was significantly longer than normal or sham operated groups (*P*<0.05 or *P*<0.01), suggesting establishment of rat model. Compared to other groups, the latency in gastrodin group at 2^nd^ test day was significantly shorter than saline group (*P*<0.05), indicating learning improvement of rat model. However, the latency in huperzine A group was observed to be longer than normal group (*P*<0.01). In spatial probe trial after 5-day place navigation trials, difference of numbers to cross the assumed platform among 5 groups was not significant (One-way ANOVA, *P*>0.05).

## Conclusion

Gastrodin may have therapeutic effect to improve learning behavior in rat model of Alzheimer's disease induced by intra-hippocampal Aβ1-40 injection by mechanism different from huperzine A.

